# A new approach to the intracardiac inverse problem using Laplacian distance kernel

**DOI:** 10.1186/s12938-018-0519-z

**Published:** 2018-06-20

**Authors:** Raúl Caulier-Cisterna, Sergio Muñoz-Romero, Margarita Sanromán-Junquera, Arcadi García-Alberola, José Luis Rojo-Álvarez

**Affiliations:** 1 Department of Signal Theory and Communications and Telematics and Computation, Rey Juan Carlos University, Camino del Molino s/n, 28943 Fuenlabrada, Madrid Spain; 2 Center for Computational Simulation, Universidad Politécnica de Madrid, Madrid, Spain; 3 Arrhythmia Unit, Hospital General Universitario Virgen de la Arrixaca, El Palmar, Murcia Spain

**Keywords:** Inverse problem, Electrophysiology, Mercer’s kernel, Laplacian, Support Vector Regression, Dual Signal Model

## Abstract

**Background:**

The inverse problem in electrophysiology consists of the accurate estimation of the intracardiac electrical sources from a reduced set of electrodes at short distances and from outside the heart. This estimation can provide an image with relevant knowledge on arrhythmia mechanisms for the clinical practice. Methods based on truncated singular value decomposition (TSVD) and regularized least squares require a matrix inversion, which limits their resolution due to the unavoidable low-pass filter effect of the Tikhonov regularization techniques.

**Methods:**

We propose to use, for the first time, a Mercer’s kernel given by the Laplacian of the distance in the quasielectrostatic field equations, hence providing a Support Vector Regression (SVR) formulation by following the principles of the Dual Signal Model (DSM) principles for creating kernel algorithms.

**Results:**

Simulations in one- and two-dimensional models show the performance of our Laplacian distance kernel technique versus several conventional methods. Firstly, the one-dimensional model is adjusted for yielding recorded electrograms, similar to the ones that are usually observed in electrophysiological studies, and suitable strategy is designed for the free-parameter search. Secondly, simulations both in one- and two-dimensional models show larger noise sensitivity in the estimated transfer matrix than in the observation measurements, and DSM−SVR is shown to be more robust to noisy transfer matrix than TSVD.

**Conclusion:**

These results suggest that our proposed DSM−SVR with Laplacian distance kernel can be an efficient alternative to improve the resolution in current and emerging intracardiac imaging systems.

## Background

Cardiac arrhythmias are often first detected with electrocardiogram (ECG) monitoring, which consists of recording the electrical potential produced by the heart in the skin [1]. ECG interpretation is crucial for arrhythmia diagnosis, and in recent years, different techniques have been studied to improve the ECG signal quality, including the search for new materials for the electrodes [2], the design of cardiac monitoring systems [3, 4] combining the analysis of ECG signals and other non-invasive signals such as the seismocardiogram [5], the estimation of fetal ECG [6], the impact of noise and improvement of signal preprocessing techniques [7, 8], or the study of ECG recognition systems for arrhythmia classification [9–11]. However, the arrhythmia treatment often requires additional bioelectric sources, and invasive methods based on catheters have been used in cardiac electrophysiology to find the arrhythmia mechanisms and to suppress them with intracardiac catheter ablation. In the last years, Cardiac Navigation Systems (CNS) have been proposed to support the conventional intracardiac mapping of the clinical procedures. These systems determine the spatial location of catheters inside the heart, record the electrical activity from the intracardiac electrograms (EGMs) in the catheter electrodes, and create electroanatomical maps of some electrophysiological feature of interest, for instance, voltage and activation time, among others. For this purpose, they sequentially measure the EGM in different locations, which extends the duration of the clinical procedure. In addition, these electroanatomical maps can be combined with anatomical information from the segmentation of medical images, such as computerized tomography or magnetic resonance [12]. However, the use of CNS has some limitations: (a) the number of EGMs required to create the map is unknown, and it is usually decided by following heuristic criteria; (b) the maps are unsuitable for unsustained arrhythmias, due to the sequential building process; and (c) the procedure can be of a long duration. Although mapping systems based on an arrays of 32 or 64 electrodes in direct contact with the heart tissue have been proposed in order to obtain a set of simultaneous EGM, their resolution is limited and depends on the simultaneous contact of the electrodes [13]. Non-contact systems have also been proposed to provide an instant endocardial or epicardial image from a floating array of electrodes [14], although these systems also exhibit limited resolution. In recent years, emerging ECG Imaging (ECGI) systems have been intensely developed, which provide us with an electrical image of the heart by projecting the bioelectrical measures obtained in the chest, abdomen, and back, onto the epicardium, using a torso model obtained from medical imaging of the patient [15]. Overall, these ECGI systems are being supported by a growing number of successful studies in the clinical practice [16], but limitations in the spatial resolution still can be present in them. Other novel clinical applications are arising from advanced uses of ECGI techniques, and among them we can find the support for the early diagnosis of arrhythmogenic right ventricular cardiomyopathy with resonance medical imaging and the help to guide the cardiac ablation of ventricular tachycardia in a completely non-invasive way [17, 18]. The inverse problem has been addressed in clinical systems from non-contact mapping systems which yielded the solved inverse problem from unipolar recordings obtained in a balloon array of electrodes [19–22]. These systems construct virtual maps with thousands of points recorded from a single catheter position and in a single beat, which is specially adequate for non-sustained arrhythmias. Still, some limitations have to be overcome, including ergonomy, volume, and manageability. Also, the virtual reconstruction needs to improve in existing systems, as well as the accuracy precision dependence on the distance (practically up to 2 cm). Also current limitations in resolution even for close regions have precluded their use in fragmented EGM analysis, and advances of sequential systems in recent years have moved the interest from non-contact mapping systems. Nevertheless, their context is still interesting and active as they continue to be the natural way to obtain an activation map with a single beat.

The electrical source estimation from regularized inversions is an ill-posed problem, thus different approaches have been proposed to deal with it [23–27]. Most of the proposed algorithms, including the truncated singular value decomposition (TSVD), the regularized Least Squares (LS), the curvature of the L-curve from Tikhonov method, and the minimum relative entropy, perform the inversion of the transfer matrix of the problem, which causes resolution reduction to their solutions [28–30]. The inverse problem has been deeply studied in many other applications, but in the case of the inverse problem in electrophysiology, the transfer matrix can be either structurally simple and only depend on the distance, or structurally more complex and include spatial information, such as the torso-organ geometries and conductivities [31–33].

In the last years, Support Vector Machines (SVMs) and kernel methods have been proposed for a variety of applications, mainly for classification and regression schemes [34–37]. Sometimes the Support Vector Regression (SVR) algorithm has been straightforwardly used to address signal processing problems on a straightforward way, this is, with little or no modification of the SVR algorithms to adapt the underlying data structure from the rich available set of signal models. Whereas this has been an advantageous approach, often the performance is affected for this algorithm mismatching to the problem. In [38], an approximation was proposed for tackling estimation problems in digital signal processing using SVMs, where a methodology to establish non-linear estimation of problems was proposed, so-called the Dual Signal Model (DSM) method, by following a similar methodology to previous SVM works addressing sparse deconvolution and non-uniform interpolation [39, 40],

In this paper, we propose to create a SVR algorithm explicitly accounting for the singularities of the data model, the physical equations and the constrains of the inverse problem in electrophysiology. For this purpose, the so-called Laplacian distance kernel is proposed for its use as Mercer’s kernel in the SVR, and the DSM is followed to create this algorithm ensuring its suitability as a single-minimum method. In general, all the statistical learning methods based on SVR have been shown to exhibit excellent regularization properties, and moreover, they allow us to readily introduce relevant *a priori* information of the problem at hand into the algorithm equations, which is a desirable advantage for this novel application.

In the current approach, we have addressed a highly simplified model problem, whereas current literature is widely devoted to more complex and realistic geometries. Nevertheless, several resolution and accuracy issues remain present in current systems, which justifies the search for new approaches starting from the very principles. It can be expected that the limitations in the regularization models used so far in the inverse problem in electrophysiology will be overcome by the deep knowledge developed in the kernel-method field during the last years, as far as the bioelectric models are rationally included in the kernel equations. On the other hand, we propose the adaptation of the non-linear DSM for SVR (DSM−SVR) algorithm to the inverse problem in electrophysiology, aiming to obtain solutions with improved resolution and regularization properties. For this purpose, we use a unipolar EGM catchment model in a homogeneous medium (inside the heart), which allows us to express the relationship between the source tissue potentials and the captured potentials registered by several distant catheters in terms of a convolutional signal model. A valid Mercel’s kernel is defined as the distance between the catheters and the cardiac tissue in the quasielectrostatic field equations. This kernel is used for the first time in the DSM−SVR algorithm, overall yielding a new algorithm for the inverse problem in electrophysiology.

The scheme rest of the paper is as follows. In Section II, the basic physical equations of a one- and two-dimensional model and the equations of the catchment model are introduced and the most usual algorithms used for the solution of the inverse problem and the DSM−SVR algorithm are also described. In Section III, results of the DSM−SVR algorithm in synthetic experiments using one- and two-dimensional models are benchmarked versus several usual algorithms. Special attention is devoted in the experiments to the effect of introducing noise in the transfer matrix instead of in the observed EGMs, which evidences the robustness of the SVM-DSP proposed algorithm. Finally, Section IV summarizes the discussion and the conclusions of the work.

## Problem formulation and methods

There exists a gap between the literature of the inverse problem in electrophysiology and the clinical obtention and use of the estimated potentials. On the one hand, the inverse problem in cardiology and in electrophysiology usually aims to estimate the measured bioelectric potential at a given anatomical point (or set of points), which is considered as equivalent to measure the potential with a unipolar electrode. Nevertheless, it is well known that the measured potential by an electrode is due to the mixed contributions of the transmembrane action potentials. Ideally, the determination of the transmembrane action potential would give the best clinical information to the electrophysiologist, hence providing a pure electrical activity and view of the arrhythmia mechanism. However, this problem has been often limited to detailed in-silico models, in which the cellular activation is generated, and then the obtained virtual potential electrograms are calculated. For this reason, we decided to work with a computational model generating the measurements in terms of the transmembrane action potentials, as this approach has been widely validated in previous works [see e.g., [41–43], despite being still far from the clinical practice.

This section describes the equations of the quasielectrostatic model used to formulate the forward problem in electrophysiology, and a simple demonstration of its expression as a Linear and Space Invariant System (LSIS) relating two multidimensional spatial signals, namely, the transmembrane potential and the extracellular potential. In order to work with this LSIS model, we implement traditional solutions to the inverse problem, including Tikhonov regularization [44–46], the modified TSVD [47], and the Total Variation (TV) method [48, 49]. Finally, we present the equations of the physical model for the forward problem by using a new formulation in terms of the non-linear DSM−SVR framework. The DSM has been previously proposed in [38, 39] for several convolutional problems in digital signal processing, such as sparse deconvolution or sinc interpolation. In this work, the DSM−SVR approach is specifically adapted to account for the quasielectrostatic conditions and for the biophysical equations of the inverse problem in electrophysiology.

### Physical model

**Fig. 1:**
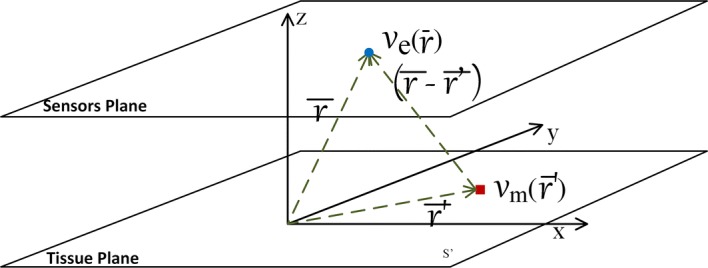
Section of two-dimensional cardiac tissue model. The upper plane corresponds to the region where the catheters are catching the measurements, whereas the lower plane corresponds to the cardiac tissue acting as bioelectric sources

We followed the mathematical modeling of the forward problem as established in the work from Ellis et al (see [50] for further details from a clinical application viewpoint). In summary, this mathematical model reformulates the volume conductor equations as a stereotypical current source, and then, the current source passes trough a filter which represents the effect of distance and impedance between the current sources and the recording electrode. In other words, the way the current source is transformed by the effects of distance and impedance to the voltage recorded at a single electrode is formulated as a convolutional operator in space. More specifically, the physical model used in this work (see Fig. 1) comprises the uptake of the extracellular potentials at a very close distance of the cardiac tissue. On the one hand, the circulating blood medium is assumed to have homogeneous conductivity. On the other hand, we take into account the source space given by a set of cells in a surface section of cardiac myocyte cells. Finally, the extracellular potential is measured at a fixed distance from these source cells. With these conditions, we can express the physical model as a spatial convolution integral, hence, the physical model can be considered as a LSIS. This formulation allows us to present a formulation in terms of the DSM framework. A preliminary approach to the proposed physical model was reported in [51].

In the present work, we consider the equations for a two-dimensional tissue as seen in Fig. 1. The lower layer corresponds to the cardiac tissue plane, with dimensions $l \times m$ (cm$^2$), and it consists of a set of cardiac cells or elements that generate an intracellular potential $v_m(\overline{r'})$, where $\overline{r'}$ denotes the position of a differential element in this source tissue plane. The upper layer corresponds to the horizontal plane where the catching catheters are situated during the electrophysiological study. Here, $v_e(\overline{r})$ corresponds to the extracellular potential captured by the catheters of all cardiac tissue cells activated in the instant $t=t_0$ at a constant height $z = z_0$ of the tissue, and $\overline{r}$ represents the general position of a differential catchment element. Note that height $z = z_0$ represents and can be seen as the radio of the metallic catheter integrating the electrical activity in its surface, in order to avoid undetermination in the mathematical equations when the catchment catheter is in contact with the tissue. Then, extracellular potential $v_e(\overline{r})$ is given by

1 $$\begin{aligned} v_{e}(\overline{r}) = c \iint _{s'} \dfrac{\nabla ^2 v_{m}(\overline{r'})}{|\overline{r}- \overline{r'}|} ds' \end{aligned}$$

where $c = a^2 \sigma _{i} / 4 \sigma _{e}$, *a* is the cell or element radio, and $\sigma _i$ and $\sigma _e$ are the intracellular and extracellular conductivities, respectively. Therefore, $|\overline{r}-\overline{r'}|$ is the distance from each point in the cardiac tissue to each point in the sensors plane [52].

In our model, we use a compact form with the following expression,

2 $$\begin{aligned} \Gamma (\overline{r}-\overline{r'}) = \dfrac{c}{|\overline{r}-\overline{r'}|} = \dfrac{c}{\sqrt{(x-x')^2 + (y-y')^2 + z_0^2}} \end{aligned}$$

so that $\overline{r'}= (x',y',0)$ are the Cartesian coordinates of the cardiac tissue source cells, and $\overline{r}=(x,y,z_0)$ are the Cartesian coordinates of the observation points in the sensor plane. Then, Eq. (1) can be rewritten as

3 $$\begin{aligned} v_{e}(\overline{r}) = \iint _{s'(\overline{r'})} \Gamma (\overline{r}-\overline{r'}) \cdot \nabla ^2 v_{m}(\overline{r'}) d\overline{r'}\end{aligned}$$

This equation represents the forward problem for the extracellular potentials as measured by the catchment catheters (see Fig. 1). With this equation, it can be readily shown that the used model satisfies the properties of linearity and spatial invariance, hence it actually represents a LSIS.

The impulse response of this LSIS can be theoretically identified in order to give an alternative model equation in terms of a spatial convolution. We can find impulse response $h_{e}(\overline{r})$ in Eq. (3) by inputing a Dirac’s delta function in the spatial domain as a transmembrane potential source, i.e., by making $v_{m}(\overline{r'}) = \delta (\overline{r'})$. In this case, the measured extracellular potential is

4 $$\begin{aligned} h_{e}(\overline{r}) = \iint _{s'(\overline{r'})} \Gamma (\overline{r}-\overline{r'}) \cdot \nabla ^2 \delta (\overline{r'}) d\overline{r'}\end{aligned}$$

By using the properties of the delta function, as shown in Appendix, we further obtain a compact expression for this impulse response,

5 $$\begin{aligned} h_{e}(\overline{r})= \iint _{s'(\overline{r'})} \nabla ^2 \Gamma (\overline{r}-\overline{r'}) \cdot \delta (\overline{r'}) d\overline{r'}= \nabla ^2 \Gamma (\overline{r}) * \delta (\overline{r}) = \nabla ^2 \Gamma (\overline{r}) \end{aligned}$$

where * denotes here the possibly multidimensional spatial convolution. We have to keep in mind that we are specifically using here $h_{e}(\overline{r})= \left. \nabla ^2 \Gamma (\overline{r}) \right| _{\overline{r}=(x,y,z_0)}$, as detailed in Appendix. Now, we can propose a forward problem which is stated as a convolutional problem, and the equation can be seen as the definition of a convolutional, spatial, and two-dimensional signal model [53], just given by

6 $$\begin{aligned} v_{e}(\overline{r}) = \Gamma (\overline{r}) * \nabla ^2 v_{m}(\overline{r}) = h_{e}(\overline{r}) * v_{m}(\overline{r}) \end{aligned}$$

This equation is valid throughout the space occupied by the source at $z=0$. Without losing the generality, for simplicity, and by working according to the model in Fig. 1, we have:

7 $$\begin{aligned} \begin{array}{lll} \widetilde{h_e}(\overline{r})= & {} \left. h_e(\overline{r}) \right| _{z=z_0} \end{array} \begin{array}{lll} \widetilde{v_e}(\overline{r})= & {} \left. v_e(\overline{r}) \right| _{z=z_0} \end{array} \end{aligned}$$

and we can finally express our fundamental equation as

8 $$\begin{aligned} \widetilde{v_e}(\overline{r})= \widetilde{h_e}(\overline{r}) * v_{m}(\overline{r}) \end{aligned}$$

Equation (6) characterizes the physical model as a LSIS in terms of the convolution operator, hence, we can use the DSM−SVR and benefit from its properties in terms of regularization and generalization capabilities from machine learning. Equation (8) continues to fulfill the convolutional relationship, and we are just accounting for the symmetry in our problem in order to address the two-dimensional signal model. Whereas these equations are general enough to support a three-dimensional model, we will restricts ourselves here to Eq. (8) for the purpose of benchmarking the generalization properties of the DSM−SVR in one- and two-dimensional models, as described later.

### Conventional algorithms in the inverse problem in electrophysiology

In order to implement and benchmark the above described equations with commonly used methods for solving the inverse problem, we need to define a discretized form of Eq. (8). For this purpose, a discretized matrix form is first defined, representing the physical model with its corresponding notation. After this, we briefly review the state of the art of the methods used to solve the inverse problem in electrophysiology that are based on this matrix.

**Fig. 2:**
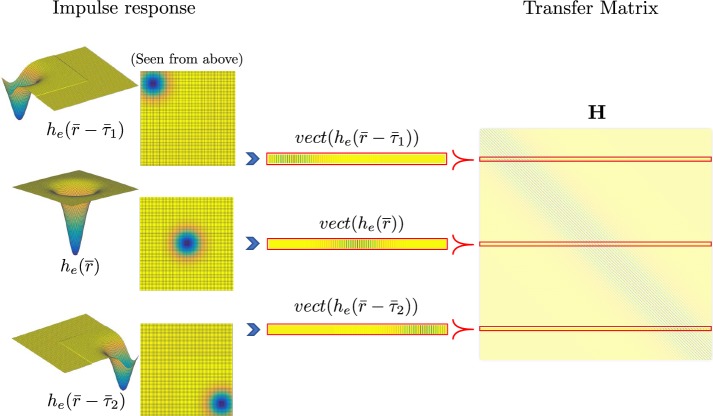
Construction scheme of transfer matrix, by using a two-dimensional uniform impulse response $h_{e}(\overline{r})$. Note that $vect(h_e(\overline{r}{-}\overline{\tau }))$ denotes a vectorization of a two-dimensional impulse response shifted from the origin by $\overline{\tau }$, where $\overline{\tau }_1$ and $\overline{\tau }_2$ are two possibly different shift factors

To obtain the solution of the forward problem, Eq. (8) can be written into its matrix form as follows,

9 $$\begin{aligned} \mathbf{v }_e= \mathbf{H \mathbf{v }_m}, \end{aligned}$$

where $\mathbf{v }_e$ and $\mathbf{v }_m$ denote the vector form for the transmembrane and for the extracellular potentials, respectively; and $\mathbf{H }$ is a Toeplitz symmetric square matrix. The size of this $\mathbf{H }$ matrix depends on the number of sensor elements and source elements on the considered planes, and it represents the convolutional matrix of the LSIS. Each of its rows is a vectorized version of a shifted two-dimensional impulse response, $h_{e}(\overline{r})$, where Eq. (5) has been previously discretized in a two-dimensional uniform grid. An example of construction of this $\mathbf{H }$ matrix is drawn in Fig. 2.

Now, the inverse problem consists of estimating the transmembrane potential while assuming $\mathbf{H }$ and $\mathbf{v }_e$ known [54–56]. The immediate solution can be expressed as $\hat{\mathbf{v }}_m= \mathbf{H }^{-1}\mathbf{v }_e$, where $\hat{\mathbf{v }}_m$ is the estimated transmembrane potential with this direct matrix inversion. Although transfer matrix $\mathbf{H }$ is assumed to be known, usually poor quality results are given by the straightforward inversion, due to the inherent ill-conditioning of the problem matrix. Hence, regularization techniques must be employed in the solution of the inverse problem. One of the most popular solutions of the inverse problem is the Tikhonov regularization [44], which is based on the LS method, and represents a standard approach in estimation analysis to the approximate solution of overdetermined systems. When the optimization objective consists of adjusting the parameters of a model function to its best fit with a data set, the LS method finds its optimum when the sum of squared residuals is minimum. Residuals are defined as the difference between the actual values of the dependent variable and the model predicted values, and in our case are given by

10 $$\begin{aligned} \hat{\mathbf{v }}_m= \arg \min _{\mathbf{v }_m}(||\mathbf{v }_e{-}\mathbf{H }\mathbf{v }_m||^{2}) \end{aligned}$$

When $\mathbf{H }$ matrix in the LS problem is ill-posed, the closed-form solution cannot be applied in a stable way, since small variations in the input data can result in large errors in the solution. A remedy often used in practice is to transform the original problem into one where the ill-posing is compensated for, by adding a term to Eq. (10), and a usual method for stabilizing this inverse solution is to use Tikhonov regularization. In this case, the expression is the following,

11 $$\begin{aligned} \hat{\mathbf{v }}_m= \arg \min _{\mathbf{v }_m}(||\mathbf{v }_e{-}\mathbf{H }\mathbf{v }_m||^{2} + \gamma ||\mathbf{R } \mathbf{v }_m||^{2}) \end{aligned}$$

and its closed-form solution can be readily shown to be

12 $$\begin{aligned} \hat{\mathbf{v }}_m= ( \mathbf{H }^T \mathbf{H }+ \gamma ^2 \mathbf{R }^T \mathbf{R } )^{-1} \mathbf{H }^T \mathbf{v }_e\end{aligned}$$

where $\gamma ^2$ is the regularization penalty parameter, which controls the weight attributed to constrain condition $||\mathbf{R } \mathbf{v }_m||^{2}$, and matrix $\mathbf{R }$ represents the regularization operator for different orders. For the method of Zero-Order Tikhonov (ZOT) solution [44], operator $\mathbf{R }$ is the identity matrix ($\mathbf R _{ii}=1$), and thus it is constrained in energy ($l_2$ norm). Now, if $\mathbf{R }$ approximates the first ($\mathbf R _{ii}=-1$ and $\mathbf R _{ii+1}=1$) or second ($\mathbf R _{ii}=-1$, $\mathbf R _{ii+1}=$2 and $\mathbf R _{ii+2}=-1$) spatial derivatives, then $\hat{\mathbf{v }}_m$ is known as the First-Order Tikhonov (FOT) [45] or the Second-Order Tikhonov (SOT) [46] solution method, respectively, and it now exhibits a smoother optimization surface gradient or curvature.

Another method used to solve this kind of inverse problems is the TSVD, which solves the following expression:

13 $$\begin{aligned} \hat{\mathbf{v }}_m= \arg \min _{\mathbf{v }_m}(||\mathbf{R } \mathbf{v }_m||), ~~ \text {subject to}~ \min _{\mathbf{v }_m}(||\mathbf{v }_e- \mathbf{H }_k \mathbf{v }_m||) \end{aligned}$$

where $\mathbf{H }_k$ is the reconstruction of $\mathbf{H }$ matrix using the *k* largest singular values. Because this method is based on a Singular Value Decomposition (SVD) process, the regularization term is defined by the number of used singular values (*k*), which is known as truncation parameter. Note that the order of $\mathbf R$ matrix also yields different solutions, such as Zero-order Tikhonov SVD (ZTSVD), First-order Tikhonov SVD (FTSVD), or Second-order Tikhonov SVD (STSVD) [47].

Another regularization method is Total Variation (TV), which is similar to Tikhonov technique, but it uses a non-quadratic norm in the regularization term, in particular, often the $\ell _1$ norm. Hence, the TV solution is obtained by solving the following problem,

14 $$\begin{aligned} \hat{\mathbf{v }}_m= \arg \min _{\mathbf{v }_m}(||\mathbf{v }_e- \mathbf{H }\mathbf{v }_m|| + \gamma ||\mathbf{R } \mathbf{v }_m||_{1}) \end{aligned}$$

where $\mathbf R$ can be also the Gradient or Laplacian operators, hence obtaining the First-order TV (FTV) [48] and the Second-order TV (STV) [49] solutions, respectively.

All these methods require an accurate determination of the regularization parameter, whose value determines the intensity and influence of the applied constraints. However, the robustness, quality, and resolution of the inverse solution are not always guaranteed because of the matrix inversion. Therefore, an alternative method is next proposed to solve the inverse problem and formulated in the simple scenarios used in this work.

### Equations for the DSM−SVR solution

The SVM are supervised learning models that are widely used to solve machine learning problems [34]. The $\nu$-SVM [57] is a well-known class of SVM algorithm used both for regression and classification. The $\nu$-SVR has been proposed for tuning insensitivity parameter $\epsilon$ in terms of a new free parameter $\nu$ with bounded range in (0, 1) [58], which makes the training process simpler. An additional class of non-linear SVR algorithms for solving convolutional data models can be obtained by considering the non-linear regression of the time instants of the observed signals, and then appropriately choosing a Mercer’s kernel. This class of algorithms is known as DSM−SVR framework [38], and in this section we present the equations used for this approach and proposed to solve the inverse problem in electrophysiology, according to the convolutional and multidimensional signal model previously developed in the preceding sections.

Be $\{[(x_i,y_i,0),v_e(x_i,y_i,z_0)], i=1,\ldots ,N\}$, such that $(x_i,y_i,0) \in \mathbb {R}^3$ is the position vector in the cardiac tissue plane of the $i^{th}$ source element, and $ve_e(\overline{r}) = v_e(x_i,y_i,z_0)$ with $z_0 > 0$, are the extracellular potential measurements of the sensor plane. The DSM−SVR uses a primal signal model that represents a regression of the independent variable in an unknown space, this is

15 $$\begin{aligned} v_e(x_i,y_i,z_0) = \langle \mathbf {w},\phi ([x_i,y_i,0])\rangle + b \end{aligned}$$

where $\phi$ is a non-linear mapping from the input space to a (usually unknown) Reproducing kernel Hilbert Space (RKHS) [36, 59], and $\mathbf {w}$ and *b* are the linear regressor and bias, respectively. Then, the formulation of the primal problem of DSM−SVR is to minimize

16 $$\begin{aligned} \small {\dfrac{1}{2}\parallel {\mathbf{w}}\parallel ^2 + C\left( \nu \varepsilon + \frac{1}{N}\sum _{i=1}^{N}(\xi _i + \xi ^*_i)\right) } \end{aligned}$$

subject to

17 $$\begin{aligned} \begin{array}{c} \langle \mathbf {w}, \phi ([x_i,y_i,0] \rangle + b - v_e(x_i,y_i,z_0) \le \epsilon +\xi _i \\ v_e(x_i,y_i,z_0) - \langle \mathbf {w}, \phi ([x_i,y_i,0] \rangle + b \le \epsilon +\xi ^*_i \\ \xi ^{(*)}_i \ge 0,\quad \forall i\ \ \epsilon \ge 0 \ \end{array} \end{aligned}$$

where $\xi ^{(*)}_i$ denotes both $\xi _i$ and $\xi ^*_i$ slack variables, *C* is the regularization parameter, and $\nu$ is an approximate ratio of the number of support vectors with respect to the number of training examples. Parameter *b* can be computed by taking into account that Eqs. (17) become equalities with $\xi ^{(*)}_i = 0$ for points with $0< \alpha ^{(*)}_i < C/N$ in the Karush–Khun–Tucker (KKT) conditions. In order to obtain the estimated extracellular potential, we solve the optimization problem as described in [34, 38, 39], and the solution takes the form

18 $$\begin{aligned} \hat{v}_e(x,y,z_0) = \sum _{i=1}^{N}(\alpha _i - \alpha ^*_i) \kappa ([x_i,y_i,0],[x_j,y_j,0]) + b \end{aligned}$$

where $\kappa (\overline{r'}_i,\overline{r'}_j) = \langle \phi (\overline{r'}_i), \phi (\overline{r'}_j) \rangle$ is a Mercer’s kernel function, and $\alpha _{i}^{(*)}$ are the Lagrange multipliers [37].

In our formulation, the kernel can be advantageously defined by using $h(\overline{r})$ according to the impulse response of the physical model in Eq. (5), which is compatible with the quasielectrostatic conditions of the cardiac tissue and while mathematically satisfying the Mercer’s condition. It is immediate to see that the following definition fulfills both, thanks to the symmetry properties of the LSIS impulse response,

19 $$\begin{aligned} \kappa ([x_i,y_i,0],[x_j,y_j,0]) = h_e(||\overline{r'}_i -\overline{r'}_j||_{1} ) \end{aligned}$$

On the other hand, Lagrange multipliers $\alpha _{i}^{(*)}$ can be assigned to correspond to the estimated intracellular potential, simply as follows,

20 $$\begin{aligned} (\alpha _i - \alpha ^*_i) = \hat{v}_{m}(\overline{r'}_i) \end{aligned}$$

Now, the solution to the physical model in terms of the DSM−SVR algorithm can be expressed as

21 $$\begin{aligned} \hat{v}_e(\overline{r}) = \sum _{i=1}^{N}\hat{v}_{m}(\overline{r'}_i) h_e(||\overline{r}_i - \overline{r'}_i||_1) = \hat{v}_{m}(\overline{r}) * h_e(\overline{r}) \end{aligned}$$

As seen, the last equation again corresponds to the convolutional form of a LSIS [60]. Therefore, by fulfilling Eqs. (19) and (20), and thanks to the symmetry of matrix $h_e(\overline{r})$, this DSM−SVR solution has been made to correspond with the same convolutional model in Eq. (8).

### Simulations and experiment design

**Fig. 3:**
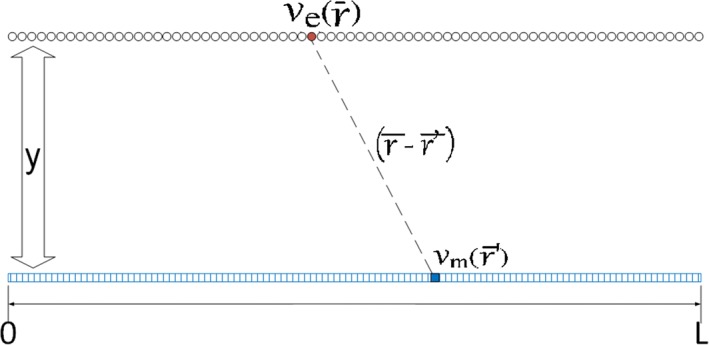
Scheme of the one-dimensional cardiac tissue model of source and catchment elements

Our tests were performed in one- and two-dimensional tissue models generated with Matlab^®^ by discretization of the continuous space model equations in "Physical model" section. For the one-dimensional case, a tissue line of elements was considered, with source elements aligned with the *x*-axis, and catchment sensors also aligned and at constant height $z_0$ (see Fig. 3). The lengths of the one-dimension cardiac-tissue line and of the catchment-sensor line were the same, hence yielding the same number of source and catchment elements after discretization, see Fig. 3. Simulations were performed with a cardiac tissue length of $L=3$ cm. The number of cells per millimeter was $N=80$ cell/cm, and we had 240 sensor elements at height $z_0$ cm. The intracellular potential was centered at the tissue origin, with depolarization starting at about − 0.5 cm and repolarization ending about 0.5 cm (see e.g. Fig. 8), which ensured that the recorded EGM included the complete depolarization and repolarization phases (spatial support was between − 1.5 cm and 1.5 cm). Note that, according to this, we did not considered source or sink EGMs, corresponding to anatomical or functional origin or end of the tissue potential propagation. The one-dimensional model of this cardiac-tissue line was the same as the one used in [51], and further details of this model can be found therein.

For the two-dimensional case, a plane tissue (see again Fig. 1) was considered with catchment sensors at coordinates (*x*, *y*) and at constant height $z_0$. We used a cardiac tissue plane with coordinates $(x',y')$ and catchment plane at height $z_0$ of the cardiac tissue. Similar to the one-dimensional case, the number of sensor elements was equal to the number of source elements, again see Fig. 1. A 3-cm$^2$ patch of cardiac tissue was symmetrically situated with respect to the coordinate origin, with 80-elements/cm$^2$ density. Accordingly, the number of sensor elements was 57, 600, all of them situated at $z_0=0.02$ cm height, but computational complexity was controlled by decimating by 2 in both spatial dimensions. This extremely high density of sensors aimed to give good quality visualization for the results of our experiments. We again considered the transmembrane potential in the center of the cardiac tissue plane to reduce boundary effects.

It can be seen from this experimental setup that the assumptions of constant $z_0$ and plane tissue and sensors are used for fundamental analysis purposes, however, a sensor array will be in general curve-shaped and with different heights for each sensor. Whereas this has clear implications on the clinical use, this spatial distortion due to non-constant height and curvature was not considered here, but rather we focused on the fundamentals of the more basic problem of plane tissue and sensors.

We benchmarked the DSM−SVR algorithm introduced in Section with several of the most traditionally used methods among those ones explained in Section. Specifically, we chose FTSVD, FOT, and FTV algorithms, and in order to optimize the regularization parameter we tested the use of two different strategies, namely, the L-Curve and the Leave-One-Out (LOO) methods. The L-Curve is supported by a graphical representation for a set of valid regularization parameters of the (semi)norm of the normalized solution versus the corresponding norm of the residuals in Eq. (11). An essential feature of the L-Curve is that the optimal regularization parameter is not far from the regularization parameter that corresponds to this L-Curve corner [61]. In the LOO method, the complete data set except one sample is used to build the model, and the out-of-sample observation is used to calculate its estimation error, so that by repeating the procedure for all the samples and averaging, the generalization error is obtained for each value of the free parameter. A similar strategy was followed for the free-parameter search of DSM−SVR, and also the L-Curve was adjusted by using LOO methods according to the Mean Absolute Error (MAE), both of them in terms of signal-to-noise ratio (SNR), when the noise was introduced in the observations, and in terms of the transfer-matrix-to-noise ratio (so-called here HNR).

## Results

In this section, we present the results obtained with the proposed DSM−SVR approach when solving the intracardiac inverse problem. A preliminary study is presented showing the ill-conditioning character of the problem with the used model and different estimation algorithms. After this, the first study was addressed in the one-dimensional model in order to determine the catchment height yielding a realistic waveform for the intracardiac EGM. Then, the following experiments were performed, both for the one- and two-dimensional models: (a) a method was scrutinized for determining the free parameters of the DSM−SVR algorithm; (b) the impact of different noise intensities was analyzed both when present on the EGM and on the transfer matrix; and (c) results were benchmarked with several methods selected among those ones that have been previously used in the inverse problem literature.

### Ill-conditioning analysis

**Fig. 4:**
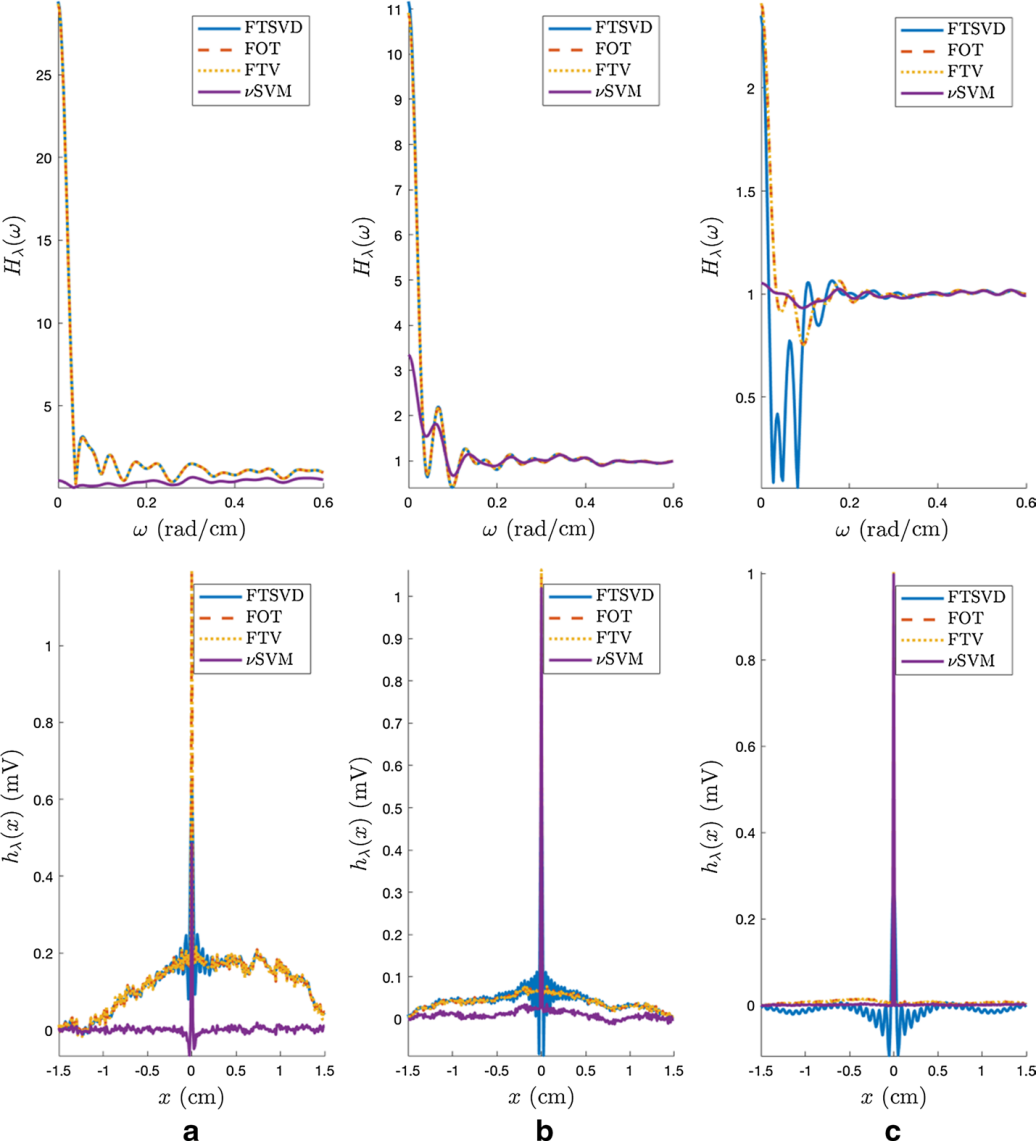
Ill-conditioning of the inverse problem in electrophysiology. A delta function is used as a source, and it is estimated in space (down) by different methods and for different SNR. The corresponding frequency responses are calculated (up):** a** SNR=15 dB;** b** SNR=25dB; **c** SNR=35 dB. Note that $H_{\lambda }(\omega )$ denotes the frequency response after tuning the free parameters on each method, and $h_\lambda (x)$ denotes the impulse response of each method

In our problem, the ill-conditioning in the transfer matrix comes from the fact that matrix $\mathbf{H}$ is built, and in essence it consists of short-delayed versions of the impulse response, i.e., if the impulse response in a single-dimensional model is given by *h*(*x*), the Toeplitz matrix consists of rows which are basically given by $h(x{-}\Delta x)$, and for small spatial displacements this implies that two consecutive raws are strongly similar. We made a numerical experiment with a single-dimensional tissue generating a spatial delta function, and then estimating it from the measurements with different methods after tuning their free parameters as later described. As seen in Fig. 4, different artifacts can be seen in the different methods, which mostly consist of a drift or leakage from the baseline and some oscillations close to the impulse activation. In frequency, this can be seen as a strong distortion in the low-frequency components of the estimated transfer function. Note that this transfer function is not just the one given by the forward problem, but also it includes the algorithm sensitivity. Accordingly, those methods which require matrix inversion are more sensitive to this effect, whereas the DSM−SVR with Laplacian kernel is built with a forward-problem formulation and the use of kernels in the formulation does not require matrix inversion in the estimation process.

### Determination of a suitable catchment height

**Fig. 5:**
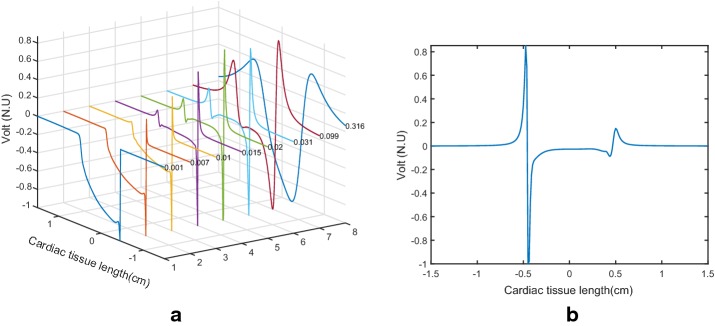
Unipolar EGM waveforms obtained with different values of $z_0$, normalized to its maximum amplitude for visualization purposes (**a**), and with $z_0 = 0.02$ cm (**b**) on the one-dimensional tissue model

Height tests were run on the sensor plane to determine the optimal height at which the signal of the extracellular potential in the one-dimensional tissue had a waveform similar to the unipolar EGMs that are usually seen in the clinical practice as a function of time in a catching electrode at a fixed position. The range of heights for $z_0$ in which the plane of sensors was varied with respect to the plane of the cardiac tissue was between 0.001 and 0.4 cm, and very different EGM waveforms were obtained throughout this range, as seen in Fig. 5a. Note that for extremely close $z_0$, the Laplacian discretization is distorted into near an inverted delta function, whereas for extremely large $z_0$ the EGM turns a far-field waveform. For intermediate values of $z_0$, there are different and intermediate distortion effects. We can check that $z_0 = 0.02$ cm in panel (b) was similar enough to the usual unipolar EGM waveform seen in electrophysiological studies. Accordingly, this was the catchment height used for all the subsequent studies, both in the one- and in the two-dimensional models.

### Free-parameter tuning strategy

**Fig. 6:**
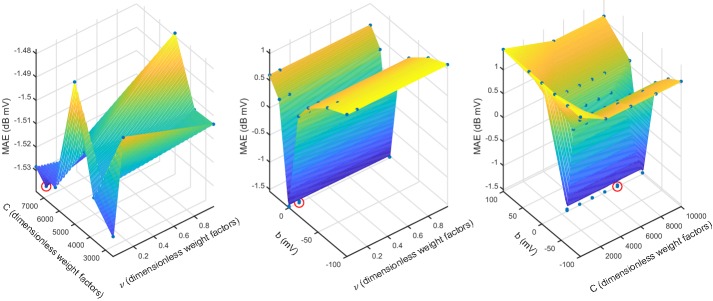
Examples of free-parameter search for DSM−SVR. Blue points indicate the set of free-parameter values that were scrutinized in the search grid. Red circles indicate the obtained optimum combination for these values, to be used in the final solution

The DSM−SVR algorithm needs to previously determine a set of free parameters, which are $\nu$, $\gamma$, *C*, and *b*. Given that these parameters are often strongly interdependent, sometimes this can represent a complex search procedure if we wanted to follow a regular grid strategy, as far as all-combined-with-all evaluation would give a extremely high amount of parameter combinations to be evaluated. Instead, we followed a non-uniform grid strategy for tuning the free parameters, as follows: (a) a lower and an upper limit were established for the possible rank of each of these four free parameters; (b) according to a previous run of searched parameters, each of them was established on either a linear or a logarithmic rank scale; (c) for each range, a middle point was established in each free parameter; (d) for all the combinations of the three values on each free parameter, a machine was trained using all the model observations except one, then the validation error was obtained for the out-of-sample observation, the process was repeated for all the observations, and out-of-sample errors were averaged; (e) the minimum error was used to reduce the range interval in the free parameters; (f) steps from (c) to (e) were repeated according to the new set of points in the grid. This procedure showed to converge in the set of observed cases. Figure 6 depicts some slice examples of the surfaces that were obtained and built with this strategy. We can see that, in general, regions with good performance correspond either to clear minimum or to flat zones, which supports the search for further specific methods to substantially reduce the computational burden associated to the free parameter search.

### Noise conditions and benchmarking

The robustness of the different algorithms was benchmarked in terms of the MAE on the two-dimensional domain. Given that we used a simulation model, we had available the actual solution of the transmembrane potential in the surface source. We added white-Gaussian noise with different power to the model in two ways. On the one hand, noise was added to the observations, i.e., to the observed extracellular potentials, as far as this is the kind of robustness more widely scrutinized in the inverse problem literature. On the other hand, noise was added to the transfer matrix, accounting for keeping its symmetry properties intact when doing so. This kind of test is less usual in the literature, however, we wanted to check for the sensitivity of the methods to the possible presence of errors in the transfer matrix errors in those scenarios when the transfer matrix is not so well-known in advance. For each method, we obtained the MAE as a function of the SNR and of the HNR. In both cases, we evaluated the performance for SNR between 0 and 70 dB.

**Fig. 7:**
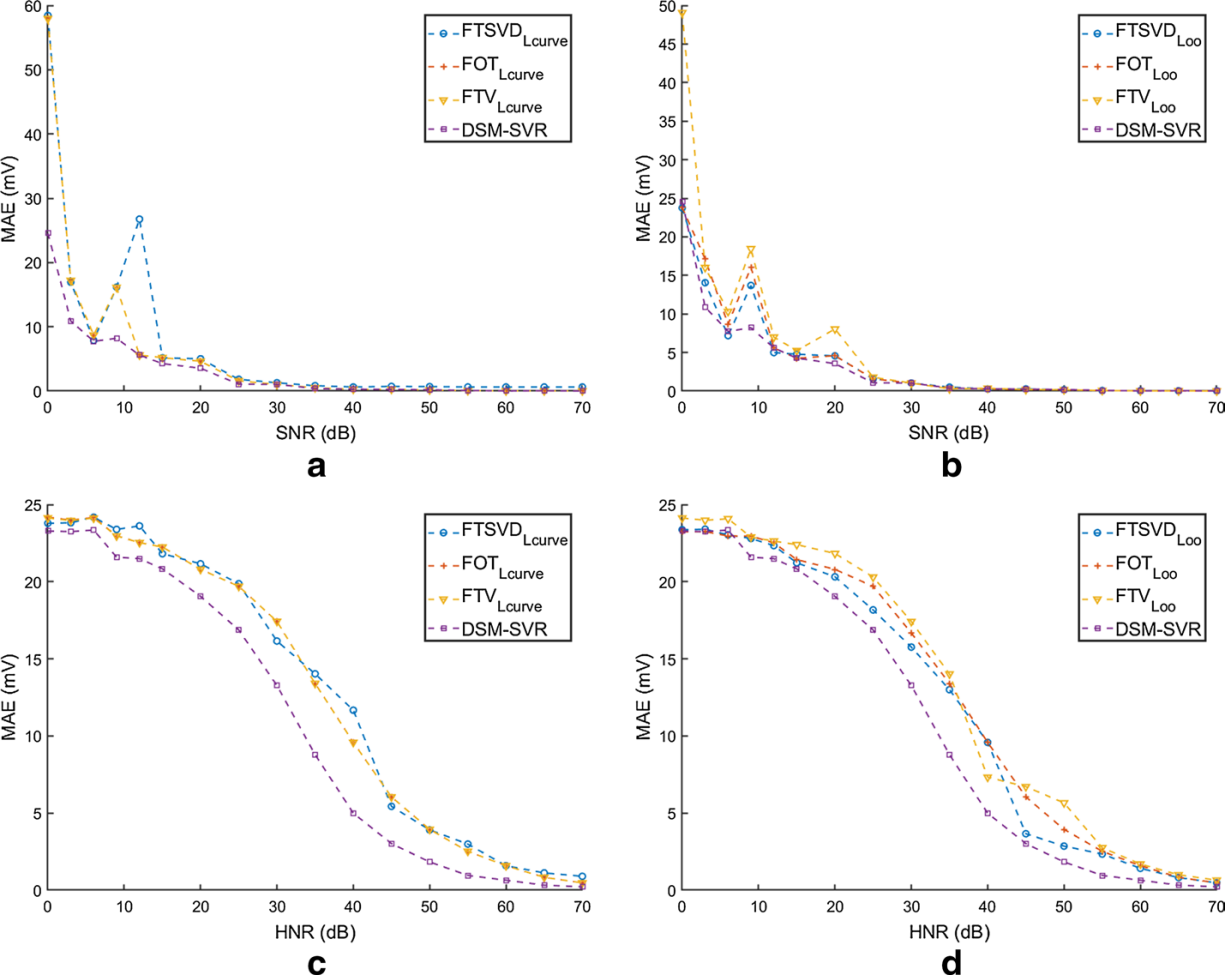
Results on noise robustness of the methods in the one-dimensional model. MAE has been calculated for all the tested methods as a function of SNR (**a**,** b**) and of HNR (**c**,** d**). We also compared the classically used L-Curve (**a**,** c**) versus the LOO (**b**,** d**) as validation criterion to tune the free parameters

Figure 7 shows the results obtained for all the used methods with SNR and HNR. On the one hand, panels (a, b) correspond to the SNR case with L-Curve and LOO, respectively. We can see that the MAE consistently decreases with increasing SNR in the catchment sensors. Also, the MAE is high for values of SNR below 5 dB, whereas it comes negligible after 30 dB. This behavior is in general consistent for all the methods, however, the DSM−SVR algorithms has a noticeably lower MAE in low-SNR conditions, and a consistently yet slightly lower MAE in high-SNR conditions. We can also see that the LOO approach tends to provide noisier MAE curves for intermediate SNR conditions in most of the classic algorithms, though it does not affect the DSM−SVR algorithm free-parameter search.

On the other hand, panels (c, d) in Fig. 7 show a different behavior of MAE as a function of HNR. First, the shape of the MAE variation is dome-like here, whereas it was exponential-like for the MAE in terms of SNR. Second, it remains at a more moderate level for low HNR values, when compared with the same range in the SNR, starting in general between 23 and 25 mV of MAE. And third, this dome-like variation is in general (and except for the range below 5 dB) above the MAE in terms of SNR. This means that these algorithms are clearly more sensitive to noise in the transfer matrix than to noise in the observations. Moreover, in the HNR plots we can see that there is a noticeable advantage of the DSM−SVR algorithm in comparison with the classical algorithms, indicating that this method is more robust when faced to perturbations in the transfer function estimation. We can finally also see also that slightly less smooth curves are obtained for the HNR in the classical methods with LOO strategy for the free parameters.

**Fig. 8:**
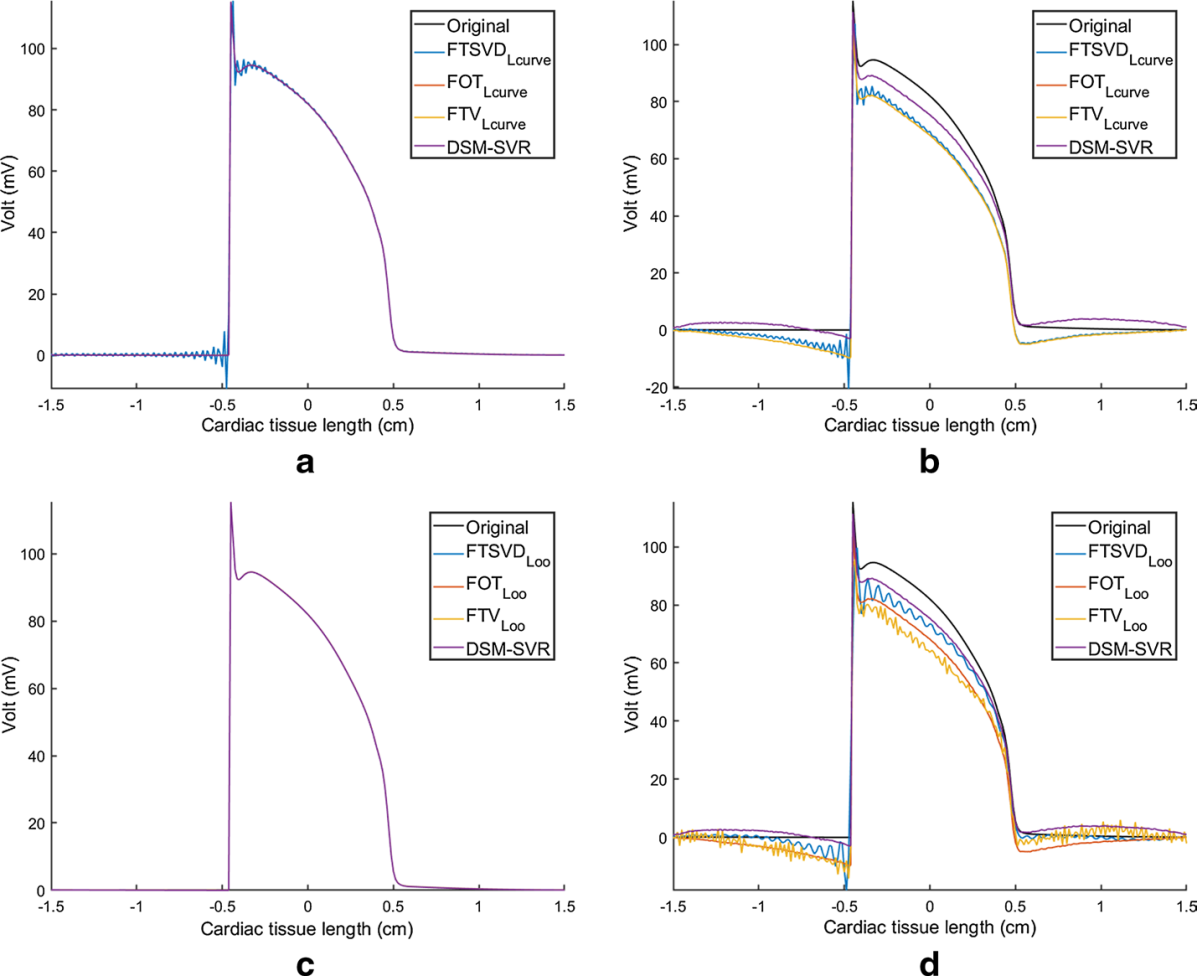
Estimated potentials in the one-dimensional source, for all the tested methods, when tuning the free parameters in the classical algorithms with L-Curve (**a**, **b**) and with LOO (**c**, **d**), without noise (**a**, **c**) and with HNR = 45 dB (**b**,** d**)

Figure 8 shows the estimated potentials in the line of source elements for the classical and for the DSM−SVR methods. Panels (a, c) show that the free-parameter estimation in the trivial case of no-noise present is clearly addressed by all the methods, though FTSVD exhibits ringing in the boundary of extremely fast variations, i.e., before and after the depolarization phase (but not in smooth variations as the repolarization phase). Panels (b, d) show the estimated transmembrane potentials for an example of intermediately high HNR of 45 dB. Overall, all the methods tend to deviate from flat regions in the presence of either sharp or smooth boundaries (depolarization or repolarization phases, respectively). Whereas DSM−SVR also exhibits this behavior, nevertheless it remains closer to the actual spatial waveform. In these conditions, LOO free-parameter search tends to give ringing solutions in the classical algorithms, whereas this is not the case for L-Curve search, though FTSVD still exhibits some ringing therein.

### Two-dimensional simulations

**Fig. 9:**
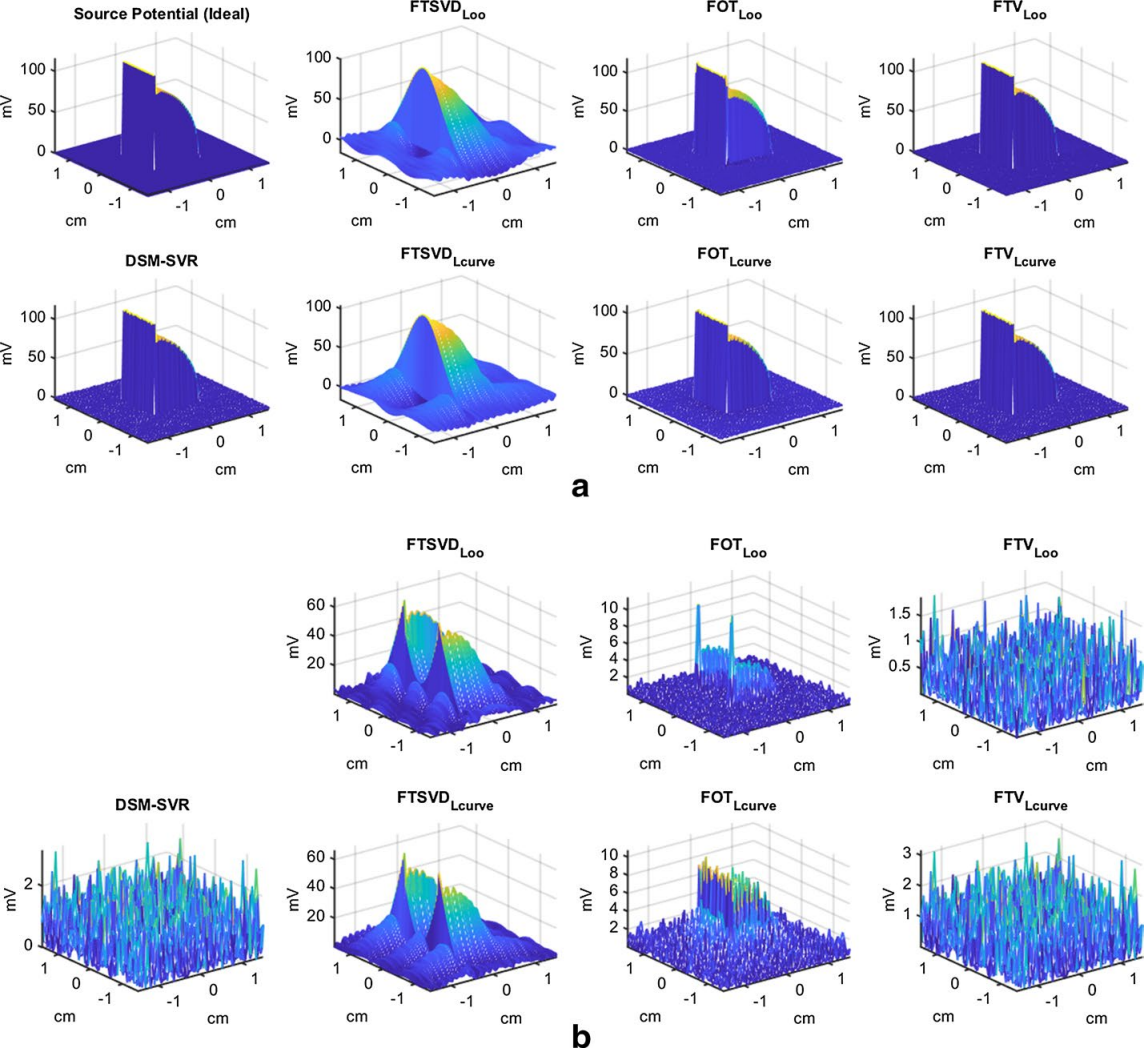
Details on the simulation results in the two-dimensional model, for SNR = 40 db.** a** Transmembrane potentials as a function of spatial coordinates, for the ideal and the estimated versions.** b** Absolute residuals for the estimated transmembrane potentials in **a**

**Fig. 10:**
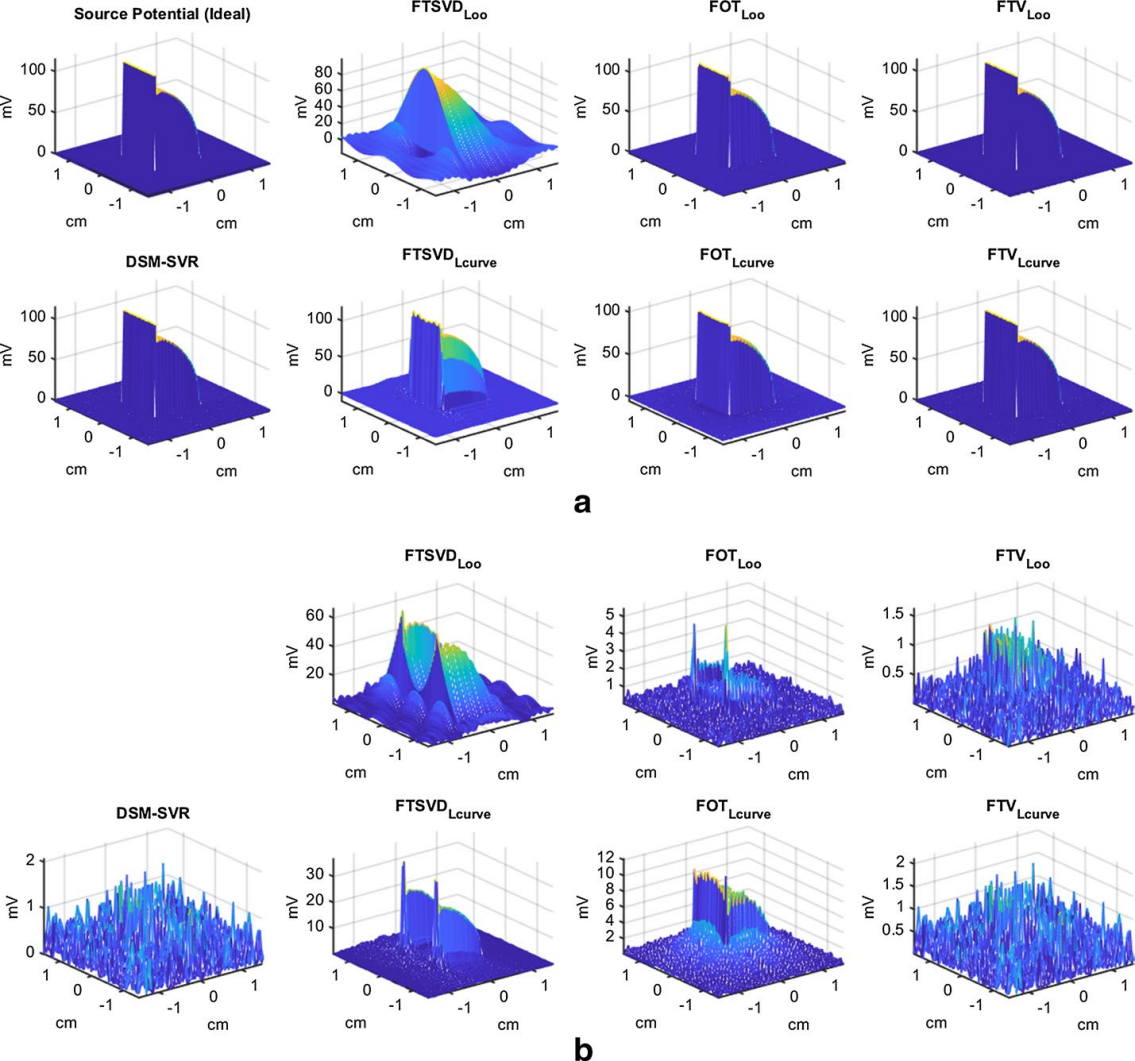
Details on the simulation results in the two-dimensional model, for HNR = 40 db.** a** Transmembrane potentials as a function of spatial coordinates, for the ideal and the estimated versions.** b** Absolute residuals for the estimated transmembrane potentials in (**a**)

Figures 9 and 10 depict some examples of the estimation behavior with the tested methods. Panel (a) shows the ideal and the estimated transmembrane potentials as a function of the two-dimensional spatial coordinates. In order to better understand the quality of the simulations, panel (b) depicts the absolute residuals as a function of the same spatial coordinates. It can be seen therein that the DSM−SVR provides a uniform distribution of the residuals with space, in contrast with FTSVD (either with LOO or with L-Curve) and with FOT (with LOO), which exhibit strong boundary effects and locally aberrant increment of the residuals. On the other hand, FOT with L-Curve yields a heteroskedastic residual distribution, which tends to increase at the inside and to reduce at the boundaries. Finally, FTV with L-Curve shows similar results to DSM−SVR, and sometimes even slightly better results when using LOO for its free-parameter tuning. Note that FTV is not the most widely used method in the current literature of the inverse problem in electrophysiology, and that its behavior with LOO free-parameter search had not been previously addressed.

**Table 1:** Results on noise robustness of the methods in the two-dimensional model

	HNR MAE (mV)					SNR MAE (mV)				
	L-Curve									
(dB)	25	30	40	50	$\infty$	25	30	40	50	$\infty$
FTSVD	1.768	1.862	1.269	0.145	1.051	2.266	1.507	8.044	0.192	1.051
FOT	3.094	2.433	1.791	1.599	1.517	4.230	2.734	1.703	1.530	1.518
FTV	1.766	1.066	0.366	0.117	8.11$e^{-12}$	4.032	2.233	0.697	0.226	8.11$e^{-12}$
	LOO									
FTSVD	7.846	7.97	8.015	8.026	8.011	8.030	8.017	8.013	8.011	8.011
FOT	1.363	0.867	0.335	0.116	4.58$e^{-13}$	1.188	0.900	0.499	0.210	4.58$e^{-13}$
FTV	1.388	0.805	0.258	0.083	7.88$e^{-12}$	2.112	1.197	0.380	0.117	7.88$e^{-12}$
	Grid search									
DSM−SVR	1.754	1.053	0.354	0.107	3.28$e^{-4}$	4.030	2.232	0.696	0.225	3.28$e^{-4}$

MAE has been calculated for all the tested methods as a function of SNR and of HNR, and also when using L-Curve versus the LOO with traditional algorithm for the free-parameter search and Grid search for DSM−SVR

Table 1 shows the MAE results for all the used methods with HNR and SNR cases using L-Curve and LOO in the traditional algorithms, and for grid search in the DSM−SVR algorithm. As can be seen, the MAE is now more similar in SNR compared with HNR, with a trend on MAE to be larger on SNR for similar noise ratios, probably due to the much larger number of observations in the two-dimensional discretized model. For the LOO tuning, the MAE tends to be smaller than for L-Curve tuning, but computational cost in the L-Curve free-parameter tuning is much smaller. On the other hand, the MAE in HNR is similar between FTV with L-Curve and DSM−SVR, the last one being slightly better in the cases with noise. The MAE in SNR exhibits similar values for FTV with L-Curve and DSM−SVR, the last one being slightly better with SNR greater than 40 dB. In the case of the LOO, the types of noise produce different MAE among methods, with the best performance reached in general by the FTV algorithm. Note that both FOT and DSM−SVR are significantly better than the other classical methods in the two-dimensional scenarios, and that the DSM−SVR algorithm is again slightly better than the FTV with L-Curve, but not so much margin can be seen now between DSM−SVR and FTV methods.

## Discussion

In recent years, research on the inverse problem in electrophysiology has been in constant development, but it is still open to improvements in its algorithms in terms of resolution and regularization. In this work, we addressed the formulation of the inverse problem using a well-known technique in the machine learning literature, specifically, the SVR following the DSM principles. This methodology had been previously used for creating digital signal processing algorithms from time-convolutional signal models. In our approach, the signal model is tackled by including in the equations (through the Mercer’s kernel and the Lagrange multipliers) the quasielectrostatic field equation conditions as *a priori* knowledge of the problem. We tested this approach in detailed simulations with synthetic experiments, using one- and two-dimensional models. Benchmarking was made with selected algorithms from classical and recent literature.

Our results showed that the inverse problem in electrophysiology is more sensitive to noise in the estimation of the transfer matrix than in the noise of the observed extracellular signals. The DSM−SVR in one-dimensional scenarios exhibited remarkable advantage with respect to all the benchmarked algorithms, specially in terms of HNR, whereas in two-dimensional scenarios it was significantly better than classical algorithms, but similar to FTV algorithms. Further research needs to be devoted to scrutinize the best (probably combined) way to tackle with high-dimensional domains. However, robustness with respect to the transfer matrix estimation seems to be a key property, which has received little attention in the literature. This advantage, mostly visible in one-dimensional simulations, probably comes from the fact that DSM−SVR does not require any kind of inversion, but its formulation rather consists of a direct problem solving and the Structural Risk Minimization Principle. In addition, it has been observed in our results that the depolarization phase is sometimes better fitted than the repolarization phase in the presence of noise. The good behavior of the algorithm in depolarization phase is likely due to its non-inversion nature, which avoids the low-pass effect implicitly associated to many regularization methods that work with matrix inversions, whereas the repolarization error could be attributed to some deviation in the free parameter search. Nevertheless, further theoretical results should be established in this setting, for providing a more solid framework when working in more complex scenarios. On the other hand, we have seen that the free-parameter tuning in the DSM−SVR can be further optimized, using techniques that require less computational cost.

In this work, we used an extremely simplified two-dimensional geometrical model. This simplification represents a conceptual approach to a more complex three-dimensional model, where symmetry properties need to be studied with further detail when establishing the method for more realistic anatomy and sensor geometry. In addition, a greater number of points in the cardiac tissue is necessary when using a three-dimensional model, and this requires a prior study of the equations for the simplified reformulation of the problem, taking into the account pre-processing information techniques. In electromagnetic problems, it is usual to follow an approach where simple problems are first scrutinized before going to more complex geometries and fields. In this setting, we have previously indicated that the plane tissue and the plane sensor geometry are not yet realistic when thinking of patient applications. However, the bandwidth, dynamics and redundancy of the spatio-temporal variations seem to point out that not a large amount of points could be necessary for reaching higher precision in the inverse problem. Our approach based on support vectors could provide with better performance in this setting, but further theoretical work has to be conducted on the effect of curvature, both in the tissue and in the sensors. From a clinical point of view, the number of sensing electrodes needs to be reasonable, as far as they will be placed on (or in) the patient on a comfortable, stable, and easy to put-and-remove way.

On the other hand, our current formulation consists on a mathematical forward model providing with the potential measured in a spatial point by a point-electrode. It is very likely that the inclusion of the shape and volume of the electrode (spherical, ring, others) can be readily included in the model, which has not yet been addressed in this work. Moreover, it is possible to think on using the method for the estimation of the transmembrane potentials for larger distances and for non-contact mapping schemes. We performed additional simulations (not included here) showing that for small changes in distance, the impulse response exhibits little (still noticeable) variation on its width, but the methods still work properly. If longer distance are scrutinized, the impulse response gets still wider, and the EGM morphology is noticeably affected. In any case, this in-silico model is not accounting for curvature effects, which could be present. Ideally, a method for estimating the impulse response would strongly improve and extend the scope of the method, which can be incorporated to the proposed method, but it has not been addressed in this work. We focused here on simple propagation, nevertheless, our view is that in fibrillatory rhythms the accurate estimation of the underlying transmembrane potentials should be strongly informative, as far as their mechanisms still remains unknown. A great amount of knowledge on fibrillatory rhythms has been established by detailed computer simulations on transmembrane potentials and ionic currents [62–64]. However, it is desirable to improve the method in more electrophysiologically simple situations before moving to complex mechanisms.

## Conclusion

The proposed approach to the DSM−SVR algorithm with Laplacean kernel can be an effective alternative in the inverse problem in electrophysiology. Our proposed DSM−SVR algorithm with Laplacian distance kernel can be an efficient alternative to improve the resolution in current and emerging intracardiac imaging systems. The sensitivity of traditional methods to noise in the transfer matrix estimation also needs to be generally taken into account by algorithms in this field.

## Appendix: Model equations

We include here the development of Eq. 5 that has been used for the demonstration of the quasielescastostatic model in see Fig. 1 corresponding to a LSIS. For this purpose, we use the properties of the one-dimensional Dirac’s delta function that can be found in [53, 65] and readily generalized to a two-dimensional domain. Equation 2 can be represented by its Cartesian coordinates as $\overline{r}= (x,y,z_0)$ and $\overline{r'}= (x',y',0)$, and then, it can be rewritten as

22 $$\begin{aligned} \Gamma (\overline{r}-\overline{r'}) = \Gamma (x^{(\prime )},y^{(\prime )},z_0) \end{aligned}$$

where $x^{(\prime )}$ denote both $(x,x')$ and of the same form can $y^{(\prime )}$ denote $(y,y')$, and similarly $v_{e}(\overline{r})$ and $v_{m}(\overline{r'})$ in Cartesian coordinates can be rewritten in our case as

$$\begin{aligned} v_{m}(\overline{r'}) &= v_{m}(x',y',0) = v_{m}(x',y') \\ v_{e}(\overline{r}) & = v_{e}(x,y,z_0) \end{aligned}$$

Now, if we replace these expressions in Eq. 3, the cardiac model can be expressed in Cartesian coordinates, as

$$\begin{aligned} v_{e}(x,y,z_0) = \displaystyle {\iint _{s(x',y')}}\Gamma (x^{(\prime )},y^{(\prime )},z_0) \nabla ^2 v_{m}(x',y') dx'dy' \end{aligned}$$

With this equation, we can prove that the cardiac model of Fig. 10 represents an LSIS. By replacing $v_{m}(x',y') = \delta (x',y')$, we have that the impulse response can be expressed as

$$\begin{aligned} \begin{array}{lll} h_{e}(x,y,z_0) &{}=&{} \displaystyle {\iint _{s(x',y')}}\Gamma (x^{(\prime )},y^{(\prime )},z_0) \nabla ^2 \delta (x',y') dx'dy' \\ &{}=&{} \displaystyle {\iint _{s(x',y')}}\Gamma (x^{(\prime )},y^{(\prime )},z_0) \left[ \dfrac{\partial ^{2} \delta (x',y')}{\partial x'^2} + \dfrac{\partial ^{2} \delta (x',y')}{\partial y'^2} \right] dx'dy'\\ &{}=&{} \displaystyle {\iint _{s(x',y')}}\left[ \Gamma (x^{(\prime )},y^{(\prime )},z_0) \dfrac{\partial ^{2} \delta (x',y')}{\partial x'^2} + \Gamma (x^{(\prime )},y^{(\prime )},z_0) \dfrac{\partial ^{2} \delta (x',y')}{\partial y'^2} \right] dx'dy'\\ \end{array} \end{aligned}$$

To solve this equation, we apply the properties of Dirac’s delta function [53, 65], which allows us to obtain

$$\begin{aligned} \begin{array}{lll} h_{e}(x,y,z_0) &{}=&{} \displaystyle {\iint _{s(x',y')}}\left[ \dfrac{\partial ^{2} \Gamma (x^{(\prime )},y^{(\prime )},z_0)}{\partial x'^2} \delta (x',y') + \dfrac{\partial ^{2} \Gamma (x^{(\prime )},y^{(\prime )},z_0)}{\partial y'^2} \delta (x',y') \right] dx'dy'\\ &{}=&{} \displaystyle {\iint _{s(x',y')}} \delta (x',y') \left[ \dfrac{\partial ^{2} \Gamma (x^{(\prime )},y^{(\prime )},z_0)}{\partial x'^2} + \dfrac{\partial ^{2} \Gamma (x^{(\prime )},y^{(\prime )},z_0)}{\partial y'^2} \right] dx'dy'\\ &{}=&{} \displaystyle {\iint _{s(x',y')}} \delta (x',y') \nabla ^2 \Gamma (x^{(\prime )},y^{(\prime )},z_0) dx'dy'\\ &{}=&{} \displaystyle {\iint _{s(\overline{r'})}}\delta (\overline{r'}) \nabla ^2 \Gamma (|\overline{r}-\overline{r'}|)d\overline{r'}= \delta (\overline{r}) * \nabla ^2 \Gamma (\overline{r}) \end{array} \end{aligned}$$

And finally, we have to keep in mind that

23 $$\begin{aligned} \begin{array}{lll} h_{e}(\overline{r}) &{}=&{} \left. \nabla ^2 \Gamma (\overline{r}) \right| _{\overline{r}=(x,y,z_0)}\\ \end{array} \end{aligned}$$

This expression allows us to obtain Eq. (5), which represents the impulse response, and allows us to represent our problem as a two-dimensional convolutional problem.
